# Retraction: Foot Loading Characteristics of Chinese Bound Feet Women: A Comparative Analysis

**DOI:** 10.1371/journal.pone.0334365

**Published:** 2025-10-13

**Authors:** The PLOS One Editors

The *PLOS One* Editors retract this article [1] due to concerns about compromised peer review. We regret that the issues were not identified prior to the article’s publication.

All authors did not agree with the retraction.
